# Effect of marital status on the survival of patients with gallbladder cancer treated with surgical resection: a population-based study

**DOI:** 10.18632/oncotarget.15476

**Published:** 2017-02-18

**Authors:** Dou-Sheng Bai, Ping Chen, Jian-Jun Qian, Sheng-Jie Jin, Guo-Qing Jiang

**Affiliations:** ^1^ Department of Hepatobiliary and Pancreatic Surgery, Clinical Medical College of Yangzhou University, Yangzhou, China

**Keywords:** gallbladder cancer, marital status, SEER, survival analysis, surgery

## Abstract

Marital status has been reported as an independent prognostic factor for survival in various cancers, but it has been rarely studied in gallbladder cancer treated by surgical resection. We retrospectively studied Surveillance, Epidemiology, and End Results (SEER) population-based data and identified 9,041 cases of gallbladder cancer with surgical treatment between 1988 and 2013. The patients were categorized according to marital status, as “married,” “never married,” “widowed,” or “divorced/separated.” Patients in the widowed group had a higher proportion of women within-group comparisons, a higher rate of white race, a greater proportion of older (≥ 60 years) patients, more frequency of adenocarcinoma, a greater number of tumors at well/moderate pathological grading, and more prevalence at the localized SEER stage, all of which were statistically significant (*P* < 0.001). Marital status was confirmed to be an independent prognostic factor by multivariate analysis (*P* < 0.001). Married patients had higher 5-year gallbladder cancer cause-specific survival than unmarried patients (*P* < 0.001); conversely, widowed patients had the lowest gallbladder cancer cause-specific survival compared with all other patients. *Conclusions* marital status is an important prognostic risk factor for survival in patients with gallbladder cancer treated with surgical resection. Widowed patients have the highest risk of death compared with other groups.

## INTRODUCTION

Gallbladder cancer (GBC) is the most common biliary tract neoplasm worldwide and is a rare but a fatal malignancy characterized by poor prognosis and absence of effective therapy [1]. Surgery is the only definitively curative treatment [2]. However, even after operation, the rate of locoregional recurrence is high. It has considerable wide geographic and ethnic variation with distinctive pockets of high incidence in Eastern and Central Europe, South and Central America, South Asia, and Japan [3, 4].

Recent literature has demonstrated that marital status is an independent prognostic factor for survival in many cancers [5–8]. Wang et al. reported that marital status was an important risk predictor in pancreatic cancer and that widowed patients were at the highest risk for cancer-specific mortality [7]. Li et al. found that widowed patients with colorectal cancer were at highest risk for death compared with other groups [8]. Few study explored the effect of marital status on GBC survival. Therefore, this study aimed to investigate the relationship between marital status and GBC survival. We selected data from the Surveillance, Epidemiology, and End Results (SEER) cancer registry to study the effect of marital status on GBC cause-specific survival (GCSS) in patients with GBC treated by surgical resection.

## RESULTS

### Baseline patient characteristics

The present study identified 9,041 eligible patients during the 25-year study period (between 1988 and 2013), including 2,453 male and 6,588 female patients. Of these, 2,549 (28.2%) were widowed, 4,632 (51.2%) were married, 1,093 (12.1%) had never married, and 767 (8.5%) were divorced/separated. Within group comparisons, the widowed group had the higher proportion of women (90.4%), white race(81.4%), older (≥ 60 years) patients (96.2%), adenocarcinoma (91.1%), and tumors at well/moderate pathological grading (50.9%) and at localized SEER stage (48.5%), all of which were statistically significant (*P* < 0.001). Table 1 showed the baseline patient demographics and tumor characteristics.

**Table 1:** Baseline demographic and tumor characteristics of gallbladder cancer patients in the SEER database

Characteristic	Total	Widowed	Married	Never married	Divorced/separated	*P*
	(*n* = 9041)	(*n* = 2549)*N* (%)	(*n* = 4632)*N* (%)	(*n* = 1093)*N* (%)	(*n* = 767)*N* (%)	
Sex						< 0.001
Male	2453 (27.1)	245 (9.6)	1760 (38.0)	283 (25.9)	165 (21.5)	
Female	6588 (72.9)	2304 (90.4)	2872 (62.0)	810 (74.1)	602 (78.5)	
Age						< 0.001
< 60	1917 (21.2)	96 (3.8)	1171 (25.3)	406 (37.1)	244 (31.8)	
≥ 60	7124 (78.8)	2453 (96.2)	3461 (74.7)	687 (62.9)	523 (68.2)	
Race						< 0.001
White	7154 (79.1)	2074 (81.4)	3664 (79.1)	812 (74.3)	604 (78.7)	
Black	844 (9.3)	203 (8.0)	324 (7.0)	197 (18.0)	120 (15.6)	
Other*	1043 (11.5)	272 (10.7)	644 (13.9)	84 (7.7)	43 (5.6)	
Year of diagnosi^s^†						< 0.001
1988–1996	1832 (20.3)	637 (25.0)	928 (20.0)	153 (14.0)	114 (14.9)	
1997–2005	3459 (38.3)	1002 (39.3)	1765 (38.1)	393 (36.0)	299 (39.0)	
2006–2013	3750 (41.5)	910 (35.7)	1939 (41.9)	547 (50.0)	354 (46.2)	
Histotype						0.027
Adenocarcinoma	8135 (90.0)	2323 (91.1)	4149 (89.6)	984 (90.0)	679 (88.5)	
Squamous cell carcinoma	93 (1.0)	32 (1.3)	43 (0.9)	10 (0.9)	8 (1.0)	
Adenosquamous carcinoma	276 (3.1)	73 (2.9)	151 (3.3)	35 (3.2)	17 (2.2)	
Other^#^	537 (5.9)	121 (4.7)	289 (6.2)	64 (5.9)	63 (8.2)	
Pathological grading						0.354
Well/moderate	4632 (51.2)	1297 (50.9)	2385 (51.5)	583 (53.3)	367 (47.8)	
Poor/anaplastic	3498 (38.7)	992 (38.9)	1774 (38.3)	409 (37.4)	323 (42.1)	
Unknown	911 (10.1)	260 (10.2)	473 (10.2)	101 (9.2)	77 (10.0)	
TNM stage						< 0.001
I/II	768 (8.5)	211 (8.3)	369 (8.0)	126 (11.5)	62 (8.1)	
III/IV	1072 (11.9)	232 (9.1)	579 (12.5)	156 (14.3)	105 (13.7)	
Unknown	7201 (79.6)	2106 (82.6)	3684 (79.5)	811 (74.2)	600 (78.2)	
Tumor size						
< 3 cm	1356 (15.0)	331 (13.0)	708 (15.3)	192 (17.6)	125 (16.3)	< 0.001
3–5 cm	891 (9.9)	232 (9.1)	450 (9.7)	108 (9.9)	101 (13.2)	
> 5 cm	524 (5.8)	125 (4.9)	268 (5.8)	81 (7.4)	50 (6.5)	
Not stated	6270 (69.4)	1861 (73.0)	3206 (69.2)	712 (65.1)	491 (64.0)	
SEER stage						< 0.001
Localized	3994 (44.2)	1236 (48.5)	1951 (42.1)	502 (45.9)	305 (39.8)	
Regional	2564 (28.4)	715 (28.1)	1328 (28.7)	294 (26.9)	227 (29.6)	
Distant	2386 (26.4)	576 (22.6)	1297 (28.0)	284 (26.0)	229 (29.9)	
Unstaged	97 (1.1)	22 (0.9)	56 (1.2)	13 (1.2)	6 (0.8)	

Abbreviations: SEER, Surveillance, Epidemiology, and End Results.

*Other includes American Indian/Alaska native, Asian/Pacific Islander, and unknown.

^†^The early and middle Year of diagnosis were all lasted nine years, the latter was lasted eight years

^#^Other cancers includes signet ring, small cell, giant and spindle cell, non-small cell carcinoma, carcinoma not otherwise specified, or undifferentiated carcinoma.

### Effect of marital status on GCSS

**Figure 1:**
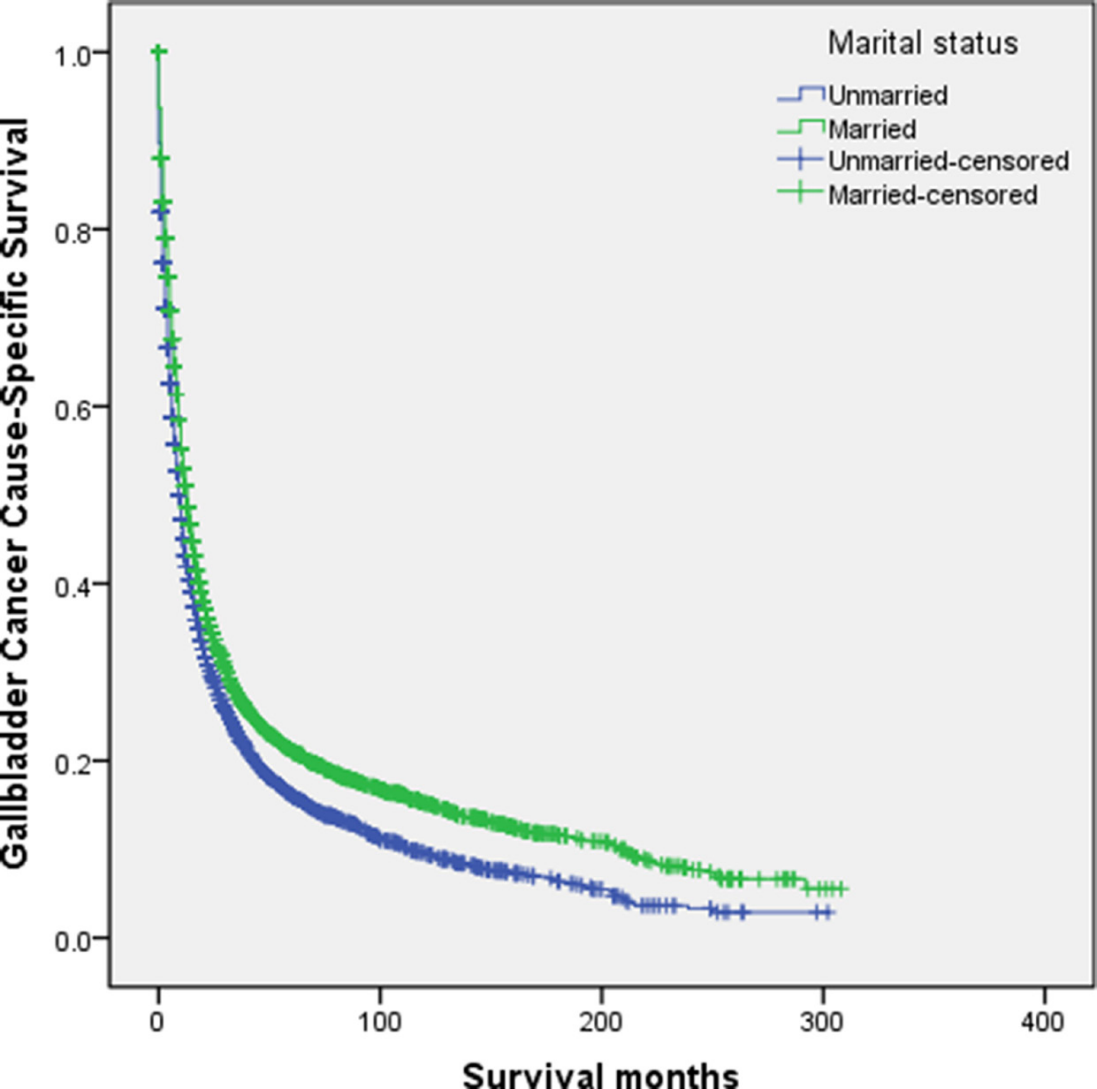
Survival curves in gallbladder cancer patients treated with surgical resection between the unmarried patients and the married patients χ^2^ = 74.829, *P* < 0.001.

**Figure 2:**
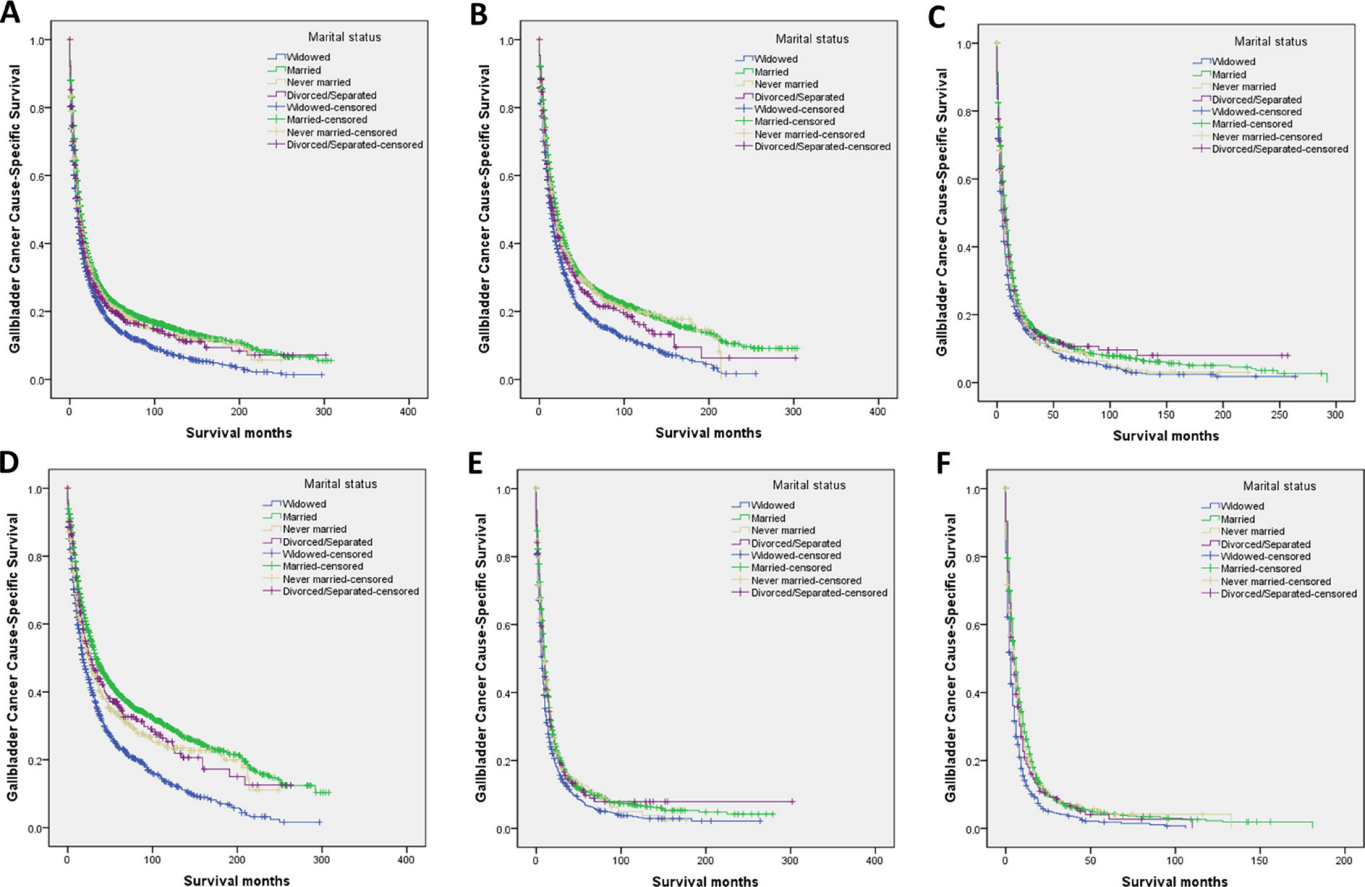
Survival curves in gallbladder cancer patients treated with surgical resection according to marital status (**A**) Overall: χ^2^ = 113.429, *P* < 0.001; (**B**) Well/moderate: χ^2^ = 69.264, *P* < 0.001; (**C**) Poor/anaplastic: χ^2^ = 37.754, *P* < 0.001; (**D**) Localized: χ^2^ = 124.013, *P* < 0.001; (**E**) Regional: χ2 = 29.216, *P* < 0.001; (**F**) Distant: χ^2^ = 68.705, *P* < 0.001.

The married group had higher 5-year GCSS than that of the unmarried patients (21.1% vs. 16.1%, *P* < 0.001) (Figure 1). The 5-year GCSS was 13.9% in the widowed group, which was the lowest compared with that in the married group (21.1%), in the never married group (20.2%), and in the divorced/separated group (18.7%); all differences were significant according to the univariate log rank test (all *P* < 0.001) (Figure 2A). Black race (*P* < 0.001), older age (*P* < 0.001), early year of diagnosis (1988–1996) (*P* < 0.001), adenosquamous carcinoma (*P* < 0.001), poor or undifferentiated pathology grade (*P* < 0.001), tumor size >5 cm (*P* < 0.001), TNM stage III/IV disease (*P* < 0.001), SEER distant stage (*P* < 0.001), and the widowed group (*P* < 0.001) were found as significant risk predictor for poor survival on univariate analysis (Table 2). When multivariate survival analysis was performed, all the aforementioned variables were validated as independent risk predictors associated with poor survival (Table 2), as follows: age (≥ 60 years, hazard ratio [HR] 1.521, 95% confidence interval [CI] 1.429–1.618), race (black, HR 1.055, 95% CI 0.973–1.144; other races, HR 0.917, 95% CI 0.855–0.994), year of diagnosis (1997–2005, HR 0.930, 95% CI 0.875–0.988; 2006–2013, HR 0.854, 95% CI 0.789–0.923), histotype (squamous cell carcinoma, HR 1.551, 95% CI 1.241–1.937, adenosquamous carcinoma, HR 1.211, 95% CI 1.064–1.378, other, HR 1.109, 95% CI 1.004–1.225), pathology grade (poor or undifferentiated tumor, HR 1.499, 95% CI 1.424–1.578, unknown pathology grade, HR 0.998, 95% CI 0.919–1.085), TNM stage (stage III/IV, HR 1.137, 95% CI 0.972–1.329; unknown stage, HR 1.259, 95% CI 1.091–1.452), tumor size (3–5 cm tumor, HR 1.123, 95% CI 1.008–1.250; >5 cm tumor, HR 1.184, 95% CI 1.044–1.344; unstated tumor size, HR 1.415, 95% CI 1.301–1.539), SEER stage (regional stage, HR 1.956, 95% CI 1.842–2.077; distant stage, HR 3.370, 95% CI 3.160–3.594; unstaged, HR 1.719, 95% CI 1.382–2.139), marital status (married, HR 0.774, 95% CI 0.732–0.817; never married, HR 0.914, 95% CI 0.842–0.994; divorced/separated, HR 0.891, 95% CI 0.813–0.977).

**Table 2:** Univariate and multivariate survival analysis for evaluating the influence of marital status on gallbladder cancer cause-specific survival in SEER database

Variable	Total	5-year CCS	Univariate analysis		Multivariate analysis	
	9041		Log rankχ2 test	*P*	HR(95%CI)	*P*
Sex			1.770	0.183		NI
Male	2453	17.6%				
Female	6588	19.1%				
Age			148.198	< 0.001		< 0.001
< 60	1917	26.5%			Reference	
≥ 60	7124	16.7%			1.521 (1.429–1.618)	
Race			19.846	< 0.001		0.032
White	7154	18.2%			Reference	
Black	844	16.9%			1.055 (0.973–1.144)	0.191
Other*	1043	23.8%			0.917 (0.855–0.994)	0.034
Year of diagnosis^†^			107.566	< 0.001		< 0.001
1988–1996	1832	15.5%			Reference	
1997–2005	3459	17.0%			0.930 (0.875–0.988)	0.019
2006–2013	3750	22.4%			0.854 (0.789–0.923)	< 0.001
Histotype			85.488	< 0.001		< 0.001
Adenocarcinoma	8135	19.3%			Reference	
Squamous cell carcinoma	93	11.9%			1.551 (1.241–1.937)	< 0.001
Adenosquamous carcinoma	276	8.1%			1.211 (1.064–1.378)	0.004
Other^#^	537	16.0%			1.109 (1.004–1.225)	0.042
Pathological grading			649.023	< 0.001		< 0.001
Well/moderate	4632	24.6%			Reference	
Poor/anaplastic	3498	9.7%			1.499 (1.424–1.578)	< 0.001
Unknown	911	23.4%			0.998 (0.919–1.085)	0.970
TNM stage			251.771	< 0.001		0.002
I/II	768	52.1%^††^			Reference	
III/IV	1072	15.3%^††^			1.137 (0.972–1.329)	0.109
Unknown	7201	23.7%^††^			1.259 (1.091–1.452)	0.002
Tumor size			226.873	< 0.001		< 0.001
< 3 cm	1356	32.7%			Reference	
3–5 cm	891	21.7%			1.123 (1.008–1.250)	0.034
> 5 cm	524	16.5%			1.184 (1.044–1.344)	0.009
Not stated	6270	16.0%			1.415 (1.301–1.539)	< 0.001
SEER stage			1911.283	< 0.001		< 0.001
Localized	3994	33.4%			Reference	
Regional	2564	9.7%			1.956 (1.842–2.077)	< 0.001
Distant	2386	3.7%			3.370 (3.160–3.594)	< 0.001
Unstaged	97	12.2%			1.719 (1.382–2.139)	< 0.001
Marital status			105.116	< 0.001		< 0.001
Widowed	2549	13.9%			Reference	
Married	4632	21.1%			0.774 (0.732–0.817)	< 0.001
Never married	1093	20.2%			0.914 (0.842–0.994)	0.035
Divorced/separated	767	18.7%			0.891 (0.813–0.977)	0.014

Abbreviations: SEER, Surveillance, Epidemiology, and End Results; CCS, cause-specific survival.

*Other includes American Indian/Alaska native, Asian/Pacific Islander, and unknown.

^†^The early and middle Year of diagnosis were all lasted nine years, the latter was lasted eight years

^#^Other cancers includes signet ring, small cell, giant and spindle cell, non-small cell carcinoma, carcinoma not otherwise specified, or undifferentiated carcinoma.

^††^3-year CCS. Because TNM stage record according to the AJCC Cancer Staging Manual (7th edition) in the SEER database began from 2009, and ended at 2013, its 5-year CCS did not exist.

NI: not included in the multivariate survival analysis.

### Subgroup analysis of pathological grading

We further analyzed the effects of marital status on survival in tumors of different pathological gradings. There were no significant differences in the subgroup of pathological gradings among the different marital status groups (Table 1), and we observed two interesting findings. On the one hand, pathological grading was an independent factor for poor survival, both in the univariate and multivariate analysis (*P* < 0.001). On the other hand, widowed patients had the lowest survival rate in comparisons at all pathological grading: For well/moderate pathological grading tumors, 5-year GCSS of widowed patients had 9.8%, 10%, and 6.4% reductions compared with that of married patients, never married patients, and divorced/separated patients respectively (all *P* < 0.01). For poor/anaplastic pathological grading tumors, widowed patients had a 3.9% reduction in 5-year GCSS compared with married patients (*P* < 0.001), a 2.3% reduction in 5-year GCSS compared with never married patients (*P* = 0.064), and a 4.2% reduction in 5-year GCSS compared with divorced/separated patients (*P* = 0.005). (Table 3, and Figure 2B–2C).

**Table 3:** Univariate and multivariate analysis of marital status on gallbladder cancer cause-specific survival based on different pathological grading

Variable	Total	5-year CCS	Univariate analysis		Multivariate analysis	
			Log rank χ^2^ test	*P*	HR(95%CI)	*P*
**Pathological grading**						
**Well/moderate**	4632					
**Marital status**			69.264	< 0.001		< 0.001
Widowed	1297	17.9%	Reference		Reference	
Married	2385	27.7%	68.612	< 0.001	0.727 (0.674–0.786)	< 0.001
Never married	583	27.9%	18.276	< 0.001	0.780 (0.695–0.877)	< 0.001
Divorced/separated	367	24.3%	7.422	0.006	0.832 (0.728–0.951)	0.007
**Poor/anaplastic**	3498					
**Marital status**			37.754	< 0.001		< 0.001
Widowed	992	7.1%	Reference		Reference	
Married	1774	11.0%	37.118	< 0.001	0.784 (0.722–0.851)	< 0.001
Never married	409	9.4%	3.437	0.064	0.891 (0.788–1.008)	0.067
Divorced/separated	323	11.3%	7.772	0.005	0.827 (0.723–0.946)	0.006

Abbreviations: CCS, cause-specific survival.

### Subgroup analysis of SEER stage

We also analyzed the effects of marital status on survival at each SEER stage. Again, we had two interesting findings. On the one hand, marital status was an independent risk factor for poor survival in patients with each SEER stage disease, both in the univariate and multivariate analysis (*P* < 0.001). On the other hand, widowed patients again had the lowest survival rate in comparisons at all SEER stages: For localized stage tumors, widowed patients had a 15.9% reduction in 5-year GCSS compared with married patients (23.5% vs. 39.4%) (*P* < 0.001), a 10.1% reduction in 5-year GCSS compared with never married patients (23.5% vs. 33.6%) (*P* < 0.001), and a 12.6% reduction in 5-year GCSS compared with divorced/separated patients (23.5% vs. 36.1%) (*P* < 0.001). For regional stage tumors, widowed patients had a 4.2% reduction in 5-year GCSS compared with married patients (6.7% vs. 10.9%) (*P* < 0.001), a 5.9% reduction in 5-year GCSS compared with never married patients (6.7% vs. 12.6%) (*P* = 0.010), and an 3.1% reduction in 5-year GCSS compared with divorced/separated patients (6.7% vs. 9.8%) (*P* = 0.015) (Table 4, and Figure 2D–2F).

**Table 4:** Univariate and multivariate analysis of marital status on gallbladder cancer cause-specific survival based on different SEER stage

Variable	Total	5-year CCS	Univariate analysis		Multivariate analysis	
			Log rank χ^2^ test	*P*	HR(95%CI)	*P*
**SEER stage**						
**Localized**	3994					
**Marital status**			124.013	< 0.001		< 0.001
Widowed	1236	23.5%	Reference		Reference	
Married	1951	39.4%	124.908	< 0.001	0.625 (0.575–0.681)	< 0.001
Never married	502	33.6%	16.809	< 0.001	0.771 (0.679–0.876)	< 0.001
Divorced/separated	305	36.1%	19.570	< 0.001	0.712 (0.611–0.830)	< 0.001
**Regional**	2564					
**Marital status**			29.216	< 0.001		< 0.001
Widowed	715	6.7%	Reference		Reference	
Married	1328	10.9%	29.036	< 0.001	0.775 (0.704–0.853)	< 0.001
Never married	294	12.6%	6.633	0.010	0.822 (0.709–0.953)	0.009
Divorced/separated	227	9.8%	5.954	0.015	0.817 (0.696–0.959)	0.013
**Distant**	2386					
**Marital status**			68.705	< 0.001		< 0.001
Widowed	576	1.9%	Reference		Reference	
Married	1297	4.4%	68.102	< 0.001	0.669 (0.604–0.741)	< 0.001
Never married	284	4.2%	19.727	< 0.001	0.716 (0.616–0.831)	< 0.001
Divorced/separated	229	4.1%	13.733	< 0.001	0.749 (0.639–0.879)	< 0.001

Abbreviations: SEER, Surveillance, Epidemiology, and End Results; CCS, cause-specific survival.

## DISCUSSION

Some studies have suggested married patients have longer overall survival and lower mortality than those who have never married, separated, widowed, or divorced for many important causes of death respectively [13–15]. By using the SEER database to determine the relationship between marital status and survival, the present study showed that married patients had significantly better GCSS than their unmarried counterparts. Widowed patients had the lowest GCSS compared with all other patients. Moreover, in multivariable analyses, the risk for widowed patients lasted even after adjusting for age, race, year of diagnosis, histologic type, pathological grading, tumor size, TNM stage,and SEER stage.

One hypothesis for the bad prognosis in unmarried individuals has delayed diagnosis with advanced tumor stage; however, in this study group, Table 1 showed the percentages of patients with well/moderate and poor/anaplastic pathological gradings were comparable among the four subgroups. Moreover, widowed patients had the highest rate of well/moderate pathological grading. Widowed patients had worse 5-year GCSS (17.9%) compared with married (27.7%), never married (27.9%), and divorced/separated (24.3%) patients (all *P* < 0.01). Similarly, among the patients with poor/anaplastic pathological grading, the widowed group had worse 5-year LCSS (7.1%) compared with married (11.0%) and divorced/separated (11.3%) patients (all *P* < 0.01). Notably, at poor/anaplastic pathological grading, there was no significant difference in GCSS between the widowed group and never married (7.1% vs. 9.4%, *P* = 0.064)—this is a result of smaller sample size.

Psychosocial factors may provide a reasonable explanation for the relationship between marital status and survival. Although psychosocial factors are regarded as an independent reason, considered separately from tumor biological characteristics and extent of treatment, these may play several important roles associated with cancer progress. Unmarried and especially widowed patients may suffer from a lack of emotional support and social attention (otherwise provided by a spouse), which contributes to more distress, depression, and anxiety than that experienced by their married counterparts [16]. Also, a cancer diagnosis can lead to distress [17]. In widowed patients, increased mortality rates may be due to the inability to relieve stress and the loss of social attention [18].

Furthermore, the level of adherence to the treatment plan may be different due to marital status. Compared with unmarried patients, married patients were inclined to be more likely to comply with treatment [19]; conversely, unrecognized clinical depression may lead to poor adherence to medical treatment and, further, that married patients showed a lower risk of major depression [20].

There is some evidence that the level of physiological stress and depression may affect cancer outcomes via different mechanisms. Increased psychological stress and decreased psychosocial support may contribute to weakened immune function and, in this way, may lead to tumor progression and mortality [21–23]. Reportedly, two meta-analyses showed that depression increased cancer mortality by 19% and 39%, respectively [24, 25]. Furthermore, perceived lack of social support has been proven to destroy the activity of natural killer cells [26]. Also, chronic stress may contribute to downregulated cortisol receptors in white blood cells [27]. This downregulation also degrades the cellular response to anti-inflammatory signals and accelerates cytokine-mediated inflammatory processes [28], which, in colorectal cancer, has been regarded as a poor prognostic factor [29, 30]. Additionally, a previous study reported that some other neuroendocrine mediators and cytokines present in depression, and stress had been associated with cancer metastasis [23]. Finally, depression and poor quality of life may lead to an increased level of vascular endothelial growth, which may promote endothelial cell migration, proliferation, and proteolytic activity [31].

The present study investigated SEER data to evaluate the relationship between marital status and the postoperative prognosis of GBC; however, the study had some potential limitations. First, the SEER database only provided marital status at diagnosis. Marital status may have changed for some patients during the therapeutic process, and these changes may have affected the outcomes. Second, some data of marital status may have been inexhaustive—for example; some married patients may have separated, while other never married patients may have been cohabitating. Third, the quality of a marriage can also affect the survival of GBC patients. Marital distress has also been associated with long-term immune consequences and has contributed to an elevated risk of various health problems [32]. Finally, the SEER GBC database lacks quality data on adjuvant therapy, comorbidities, and recurrence.

To our best knowledge, this is the first report studying the effect of marital status on the survival of GBC patients treated with surgical resection. Despite these potential limitations, our study confirmed that unmarried patients are at greater risk for cancer-specific mortality. Furthermore, we showed that widowed patients were always at the highest risk for death via cancer. Psychosocial factors may be the primary reasons leading to poor survival in unmarried patients. Therefore, to improve postoperative survival, physicians should take into consideration social supports during their care of unmarried patients with GBC and especially of widowed patients. Further clinical trials should be performed to confirm our hypothesis.

### Statistical analysis

We analyzed sex, age, race, primary tumor site, histologic type, pathology grade, tumor size, TNM stage, SEER stage, survival months, vital status, and marital status at the time of diagnosis. The TNM stage according to the criteria described at the American Joint Committee on Cancer (AJCC) Cancer Staging Manual (7th edition) was established. We categorized patients as “never married,” “married,” ”widowed,” or “separated/divorced.” “Unmarried” included “never married,” “widowed,” and “separated/divorced.” The individuals who were separated and who were divorced were grouped together in the group in our study.

The primary focus of this study was GCSS, which was obtained from the date of diagnosis of gallbladder cancer and the date of gallbladder cancer cause-specific death. Deaths attributed to GBC were treated as events, and deaths from other causes were treated as censored observations.

The baseline patient demographics and tumor characteristics were analyzed using the chi-square test. Death rate of the GBC was evaluated between groups using the Kaplan–Meier method. Risk factors for survival outcome were assessed using multivariable Cox regression models. All statistical analyses were performed using the statistical software package SPSS 22.0 software (IBM Corp, Armonk, NJ, USA). A *P* value < 0.050 was considered statistically significant.

## MATERIALS AND METHODS

### Baseline patient characteristics

The SEER program of the National Cancer Institute is an authoritative source of information on cancer incidence and survival in the United States. The SEER program registries routinely collect patient clinical data including demographics, the tumor morphology and stage at diagnosis, first course of treatment, the follow-up for survival, and so on. SEER currently collects and publishes cancer incidence and survival data from 18 population-based cancer registries that represent approximately 30% of the population in the United States.

SEER data contain no identifiers and have been widely used for studies of the relationship between marital status and survival outcome in patients with cancer [5,6,9–12]. We used SEER*Stat 8.1.5 software to identify patients with a histopathologic diagnosis of GBC between 1988 and 2013. SEER registry patients eligible for this cohort included those with the following histologic type ICD-O-3: adenocarcinoma (8140, 8141, 8143, 8147), papillary adenocarcinoma (8260, 8261, 8262, 8263), mucinous adenocarcinoma (8480, 8481), adenocarcinoma with metaplasia (8571, 8572, 8573, 8574, 8575, 8576), papillary carcinoma (8050, 8051, 8052), duct carcinoma (8500, 8501, 8503, 8504, 8507, 8508), squamous cell carcinoma (8070, 8071, 8072, 8073, 8074, 8075, 8076, 8078), adenosquamous carcinoma (8560, 8562), or other cancers, including signet ring (8490), small cell (8041, 8043), giant and spindle cell (8030–8035), non-small cell carcinoma (8046), carcinoma not otherwise specified (8010, 8011, 8012, 8013, 8014, 8015), or undifferentiated carcinoma (8020, 8021, 8022). Patients with any other histologic type were excluded from analysis.

We excluded patients who were less than 18 years at diagnosis; did not undergo surgical resection for GBC; had multiple primary cancers, of which the GBC was not the first; and who had an unknown cause of death or unknown survival length.

According to the SEER staging system, tumors that remained *in situ* or confined to the organ of origin were considered to be localized; tumors that invaded locally or metastasized to regional lymph nodes were regarded as regional, while those that traveled to distant organs were considered to be distant.
